# A novel poly(3-hydroxybutyrate-*co*-3-hydroxyvalerate) (PHBV)-PEG-melatonin composite scaffold enhances for inhibiting bone tumor recurrence and enhancing bone regeneration

**DOI:** 10.3389/fphar.2023.1246783

**Published:** 2023-08-17

**Authors:** Wei-Lin Zhang, Zhi-Wen Dai, Si-Yuan Chen, Wei-Xiong Guo, Zhong-Wei Wang, Jin-Song Wei

**Affiliations:** Department of Spinal Degeneration and Deformity Surgery, Affiliated Hospital of Guangdong Medical University, Zhanjiang, China

**Keywords:** PHBV, PEG, melatonin, scaffold, osteosarcoma, bone tissue regeneration

## Abstract

**Introduction:** Postoperative comprehensive treatment has become increasingly important in recent years. This study was to repair tissue defects resulting from the removal of diseased tissue and to eliminate or inhibit the recurrence and metastasis of residual tumors under the condition of reducing the systemic side effects of chemotherapeutic drugs. To address these challenges, multifunctional scaffolds based local drug delivery systems will be a promising solution.

**Methods:** An optimal drug-loaded scaffold material PHBV-mPEG5k (PP5) was prepared, which is biocompatible, hydrophilic and biodegradable. Furthermore, this material showed to promote bone healing, and could be conveniently prepared into porous scaffold by freeze-drying the solution. By means of introducing melatonin (MT) into the porous surfaces, the MT loaded PP5 scaffold with desirable sustained release ability was successfully prepared. The effectiveness of the MT loaded PP5 scaffold in promoting bone repair and anti-tumor properties was evaluated through both *in vivo* and *in vitro* experiments.

**Results and Discussion:** The MT loaded PP5 scaffold is able to achieve the desired outcome of bone tissue repair and anti-bone tumor properties. Furthermore, our study demonstrates that the PP5 scaffold was able to enhance the anti-tumor effect of melatonin by improving cellular autophagy, which provided a therapeutic strategy for the comprehensive postoperative treatment of osteosarcoma.

## 1 Introduction

Bone tumors, particularly osteosarcoma, have a higher incidence in adolescents. The current treatment for metastatic bone cancers involves the resection of tumor tissue, combined with chemotherapy and radiotherapy, unfortunately, this approach often results in bone defects (Ma et al., 2017). Therefore, there is a need for a multifunctional method that can eliminate residual cancer cells while repaire bone defects (Xue et al., 2020). Local drug delivery systems using multifunctional scaffolds can effectively address these challenges. Yang et al. (2020) demonstrated the efficacy of a PDA-coated, DOX-loaded LHAp/PLGA fibrous scaffold, in achieving controlled drug release, inhibiting tumor cell growth and promoting the proliferation of normal cells during the early stages of treatment (Lu et al., 2021).

The copolymer of 3- hydroxybutyrate and 3-hydroxyvalerate (PHBV) is a piezoelectric a material that possesses extremely good biocompatibility has been used in tissue engineering and regenerative medicine for many years (Ye et al., 2018; Chen et al., 2021; Kaniuk,, Stachewicz, 2021). In addition to biocompatibility, the copolymer PHBV has excellent biodegradability. And the piezoelectric properties of PHBV result in a negatively charged surface potential on material’s surface, which is similar to that of human bone. And recent studies have demonstrated the beneficial impact of negative potential on the surface of materials, particularly in terms of promoting mineralization and osteogenic differentiation of cells (Kang et al., 2013; Mihaila et al., 2014). As a result, PHBV has emerged as the most promising biopolymer for a variety of applications, particularly in bone tissue engineering where it has been extensively utilized (Huang et al., 2010; Jacob et al., 2018).

However, due to its hydrophobic characteristic, causing low cellular interaction and bioactivity, which limits the application of PHBV in bone regeneration. Polyethylene glycol (PEG) with biodegradable and biocompatible and commercially available in molecular weights ranging from 500 to 20,000 Da, which has been approved by the US Food and Drug Administration (FDA) for using in human body, is the most commonly used hydrophilic component of polymeric micelles. To make up the shortcomings of PHBV, a new block copolymer (PHBV-mPEG5K, PP5) was developed using mPEG with an average molecular weight of 5,000 and PHBV, the hydrophilicity, biocompatibility and biodegradability of PP5 were improved compare to PHBV, making it a promising candidate for constructing tissue engineering scaffold. Combining the above, PP5 was employed as a carrier for melatonin to repair bone defect and prevent tumor recurrence after osteosarcoma surgery.

Melatonin (N-acetyl-5-methoxy-tryptamine) plays a significant role in regulating the sleep-wake cycle, declining tumor progress and ameliorating immune system actions (Ekmekcioglu, 2006; Cheng et al., 2013). Some Studies revealed that melatonin can be effective against many types of tumors including Endometrial Cancer and osteosarcoma et al. (Dana et al., 2020; Yang et al., 2022). In addition, melatonin levels are related to bone metabolism which can prevent bone degradation and promote bone formation (Cutando et al., 2012). The results of studies indicate that melatonin may have anti-tumor properties by activating or inhibiting various mechanisms including apoptosis, anti-proliferative and anti-oxidant activities. Accumulating evidence studies has provided evidence of melatonin’s anti-osteosarcoma properties, making it a promising candidate as an adjuvant agent in osteosarcoma treatment. Melatonin is a safe potential therapeutic agent for children and adolescents and has shown promise as an adjuvant in reinforcing the therapeutic effects of chemotherapies while also abolishing the unwanted consequence.

In this study, we present a new approach for MT delivery using a MT-loaded PP5 scaffold. The PP5 scaffold was prepared by modifying PHBV with mPEG5k and lyophilizing the solution. MT was then loaded onto the scaffold by dissolving it with GelMA and coating to the scaffold porous surfaces. GelMA has been broadly applied as biomaterials for bone tissue regeneration and other tissue repair owing to suitable biological properties and tunable physical characteristics (Sun et al., 2018). Especially, the source of GelMA is collagen, the major component of ECM, so there is much arginine-glycine-aspartic acid (RGD) sequences in GelMA that are favorable for cell adhesion, migration and growth (Liu and Chan-Park, 2010; Klotz et al., 2016). We characterized the chemical structure of PP5 and evaluated the drug release behavior, bioactivity to promote bone defect repair and ability for preventing bone tumor of the MT-loaded PP5 scaffold. Finally, the mechanism of the enhancement of MT’s antitumor effect by PP5 scaffold was also revealed.

## 2 Methods and materials

### 2.1 The preparation of PHBV-mPEG5k (PP5) scaffold

To prepare PHBV-mPEG5k (PP5), mPEG-5k (Sigma, China) was dissolved in dry dichloromethane. The solution was then heated to 80°C under vacuum for 24 h and subsequently cooled to room temperature. The mPEG-5k solution was then added dropwise to the hexamethylene diisocyanate solution with stirring and the mixture was left to react in the dark at room temperature for 6 h to obtain mPEG5k-NCO. Next, PHBV (Tianan Biologic Material Ltd., China) was dissolved in dry dichloromethane and heated to 80°C under vacuum for 5 h and subsequently cooled to room temperature. The mPEG5k-NCO solution was then added dropwise to the PHBV solution and the mixture was left to react in the dark at room temperature for 3 days. The crude product was obtained by rotary evaporation to remove the dichloromethane. The resulting product was then purified by dissolving it in tetrahydrofuran and gradually adding distilled water with stirring. After filtration and washing with distilled water, the purified product was dried under vacuum to obtain PHBV-PEG5k (PP5).

For preparation of PP5 scaffold, PP5 was dissolved in deionized water and heated to 50°C for 24 h, until the polymer was completely dissolved, transferring the solution to 6 mm cell culture dish and stored at −20°C for 12 h. Finally, the solution was lyophilizated at −20°C, the scaffold was obtained.

### 2.2 Characterization of PHBV-PEG5k (PP5)

The Fourier transform infrared spectra of mPEG5k, PHBV and PP5 were recorded using a Perkin Elmer spectrophotometer (spectrum 2) through the solid-state KBr pellet method within the range of 4,000–400 cm^−1^.

The ^1^H-NMR of the polymer was recorded using FT-NMR JEOL AL 500 FT-NMR Proton nuclear magnetic resonance spectrometer in CDCl_3_. Tetramethylsilane was used as an internal reference.

### 2.3 Melatonin loading on the scaffold

A series of GelMA (SunP Gel G1) gels with varying concentration (0.25% w/v, 0.5% w/v, 1% w/v, 1.5% w/v and 2% w/v) were prepared to find the optimal concentration for loading MT. A mixture of 0.05 gMT and different masses of GelMa were added to 10 mL Phosphate Buffer Saline (PBS), and stirred at 650 r/min for 2 h in a 40°C water bath until fully dissolved. Then the MT solution was fully absorbed by PP5 scaffold and placed at room temperature (23–25°C) until it underwent liquid-solid conversion, resulting in the production of MT-loaded PP5 scaffold.

### 2.4 Determination of loading efficiency (LE) and loading capacity (LC)

To prepare the MT solution, a mixture of absolute ethanol and chloroform (V: V = 9:1) was used as the solvent. The maximum absorption peak was measured using an ultraviolet-visible spectrophotometer (Mettler Toledo, Switzerland) within a wavelength range of 200–350 nm. A standard curve was drawn by fitting the absorbance with the mass concentration of the MT solution at the wavelength of the maximum absorption peak (278 nm) using linear regression. Freeze-dried MT-loaded GelMa gel (50 mg) was dissolved in PBS (10 mL) and centrifuged at 12,000 rpm for 5 min. Then, 0.5 mL of the supernatant was diluted in PBS in a 25 mL volumetric flask and the absorbance value of the solution was measured at 278 nm. The mass of melatonin was calculated from the standard curve and the LE and LC was calculated by following formula: *In vitro* scaffold drug release study

$$LE=m_{a}/m_{b}\times 100\%;LC=m_{a}/m_{c}\times 100\%$$

where m_a_ means the mass of melatonin in MT-loaded GelMA gel, m_b_ represents total mass of MT and m_c_ is the mass of GelMA in MT-loaded GelMA gel.

### 2.5 *In vitro* drug release behavior

Standard curve of MT was first drawn using PBS (pH = 7.4) as solvent under the wavelength of the maximum absorption peak (278 nm). MT-loaded GelMA (100 mg) was dissolved in PBS and placed in a dialysis bag. The dialysis bag was then placed in a beaker containing the same pH PBS buffer and was vibrated horizontally in a constant temperature shaker (37°C ± 0.5°C) with a vibration frequency of 50 times/min. The release liquid (5 mL) was replaced with same amounts of fresh PBS at selected time intervals. The absorbance value of the solution was measured at 278 nm and the drug release rate was calculated as follow:

$$R\left(\%\right)=\frac{\rho_{n}V+V_{i\sum_{t}^{n-i}\rho i}}{M_{D}}\times 100$$

where R means the cumulative drug release rate (%); *n* means the number of samplings. (%) represents the mass concentration (g/L) of the drug in the nth release. V represents the total volume of the release liquid. $\rho_{i}$ represents the mass concentration (g/L) of the drug in the ith release, V_i_ represents the volume of the release liquid at the ith sampling and MD means the mass of drug loaded (g).

### 2.6 Biocompatibility tests

Human bone mesenchymal stem cells (hMSCs) were originally purchased from Cell Bank of Shanghai Institutes for Biological Sciences. The PHBV and PP5 scaffold were sterilized by ultraviolet irradiation for 30 min and then placed in a 48 well cell culture plate. The hMSCs were seed on both materials at a density of 5 × 10^4^ cells/well, incubated at 37°C, 5% CO_2_ atmosphere and 95% humidity for predetermined durations of 24 h, 48 h and 72 h. Then the materials with adhered cells were rinsed with PBS, stained with phalloidin and 4,6-Diamidino-2-phenylindole dihydrochloride (DAPI) for 30 s, and finally observed under inverted microscope.

In order to quantitatively evaluate the cell viability on both materials, CCK8 assay was used. Following incubation as described above, 10 ul CCK8 was added to each well and the plate was incubated in darkness at 37°C for 4 h, the absorbance (OD) values were measured at 450 nm (iMark, Bio-rad, USA).

### 2.7 Evaluation of osteogenic differentiation of PP5 scaffold *in vitro*

The mRNA expression of collagen I (COL I), Runt-related transcription factor 2 (Runx2), osteocalcin (OCN), osteoprotegerin (OPG) and alkaline phosphatase (ALP) were quantified to assess the osteogenic differentiation of different scaffolds by Real-Time PCR. hMSCs were adhered in 6-well plates and RNA were harvested after osteogenic induction for 7 days using TRIzol reagent (Invitrogen). Prime Script RT reagent kit (Takara, Shiga, Japan) was used for mRNA to be reversely transcribed into complementary DNA. ABI 7,900 was used for quantitative analysis of the reverse transcription reaction. The data were normalized to glyceraldehyde-3-phosphate dehydrogenase (GAPDH) expression and analyzed by the 2^−ΔΔCT^ method.

Alizarin red S staining was used to evaluate the extracellular calcium deposition, revealing individual osteo-induction capacity of different scaffolds. HBMSCs were seeded into 24-transwell plates and co-cultured with PHBV or PP5 in osteogenic differentiation medium for 21 days. After 21 days, cells were washed twice with PBS and fixed with 4% paraformaldehyde for 15 min and then stained with Alizarin Red S (2% aqueous, Sigma) solution for 30 min.

### 2.8 *In vivo* bioactivity analysis

Femur defect models were established on sixteen 8-week-old male SD rats to examine the repair efficacy of the PP5 scaffolds. The femoral defects model was prepared according to previously described protocols (Chou et al., 2008). The experimental rats underwent general anesthesia with intraperitoneal injection of 50 mg/kg pentobarbital sodium All animals were cared and experiments were conducted in accordance with the National Institutes of Health Guide for the Care and Use of Laboratory Animals (2011 revision). The experimental protocol was approved by the Ethics Committee of Affiliated Hospital of Guangdong Medical University (No. PJKT 2022-046). All possible efforts were made to reduce the number of rats used and discomfort to the rats. Afterwards, the surgical sites were shaved and disinfected. A bone defect of approximately 4 mm in diameter and 5 mm in depth was created at the right femoral epicondyle using a slow-speed cylindrical burr. Subsequently, according to the type of scaffolds implanted, the animals were randomly divided into two groups (8 rats per group): (1) no treatment (defect control), (2) PP5-scaffold. The post-operative pain was controlled by intra-muscular injection of 5 mg/kg morphine every 4 h. Two months post-implantation, the rats were sacrificed and the femurs were extracted for micro-CT analysis. 3D bone reconstruction and quantitative analysis of bone regeneration were conducted using the system software. Bone volume/tissue volume (BV/TV) and bone mineral density (BMD) of each sample were analyzed and calculated. Afterwards, the collected femur samples were decalcified and prepared for hematoxylin-eosin (H&E) staining to visualize bone regeneration.

### 2.9 Evaluation of anti-tumor migration effect

Transwell system was used to analyze cell migration, cells were seeded in the top chambers of the transwell migration plates in DMEM containing 10% FBS. At 24 h after seeding, cells that migrated to the underside of the trans well were stained with 0.5% crystal violet for 5 min and imaged in ten random fields. To investigate the invasion and migration of cells in PP5 or MT-loaded PP5 scaffold, the cells (5 × 10^5^) were injected to the scaffold with a designated site, and the cell migration away from the injection sites was monitored with a light microscope (Leica, Germany) and quantified using ImageJ.

### 2.10 Evaluration of anti-tumor performance *in vivo*

The protocols of use and care of the animals were reviewed and approved by Institutional Animal Care and Use Committee of Affiliated Hospital of Guangdong Medical University. 2.0 × 10^5^ LM-8 (murine osteosarcoma cells) per mouse were injected subcutaneously into the leg of the nude mouse (6 week-old Balb/c, Vital River Laboratory Animal Technology Co. Ltd., Beijing, China). When the volume of the tumor grew to about 300 mm^3^, the mice were randomly divided into three groups (*n* = 4). A small incision was made on the leg of the mouse and the functional cuboid scaffold (length: 8 mm, width: 2 mm, height: 2 mm) was implanted under the tumor. The first day of the treatment was regarded as Day 0. The weight of tumor was measured after 1 month. The tail vein injection was used for MT group treatment, and the dose of MT was consistent with that of MT + PP5 group.

### 2.11 Mechanism exploring about enhancement of anti-tumor effect by PP5

Western blotting was used to determine the protein expression level of LC3 II/LC3 I and P62/GAPDH.The cellular proteins were extract with by cell lysis buffer (Invitrogen, United States). Protein samples were separated on 10% SDS-PAGE, followed by transferred onto PVDF membranes (Millipore, United States). The membranes were blocked with 2% bovine serum albumin (BSA) in PBST for 1 h at room temperature, then the membranes were incubated with the primary antibodies and secondary antibodies for 1 h at 37°C. Finally, the membrane was observed by sensitive enhanced chemiluminescence (ECL) detection kit (Beyotime, China) and bands were detected by ChemiDoc Imaging Systems (Bio-Rad, United States).

The immunocytochemistry was performed on cells grown on 22 × 22 mm coverslips. Wash the cells with PBS for 3 × 5 min. Fix the cells on a coverslip and fix them with 3.7% formaldehyde in PBS for 5 min. Permeabilize cells with PBS containing 0.1% Triton X-100 for 3 min. Block with 7.5% BSA in PBS. Incubate with primary antibodies diluted in PBS containing 0.5% BSA. Wash the cells 3 times. Incubate with secondary antibodies for 1 h. Counterstain the nuclei with PBS containing 10 ng/mL Hoechst 33342 for 3 min. Fluorescence is observed under a microscope.

### 2.12 Statistical analysis

Statistical data analysis was performed with SPSS software. Data from at least three independent experiments were collected, analyzed, and expressed as mean ± standard deviation (SD). Results with *p*-values of < 0.05 were considered statistically significant.

## 3 Results

### 3.1 Synthesis and characterization of PP5

**FIGURE 1:**
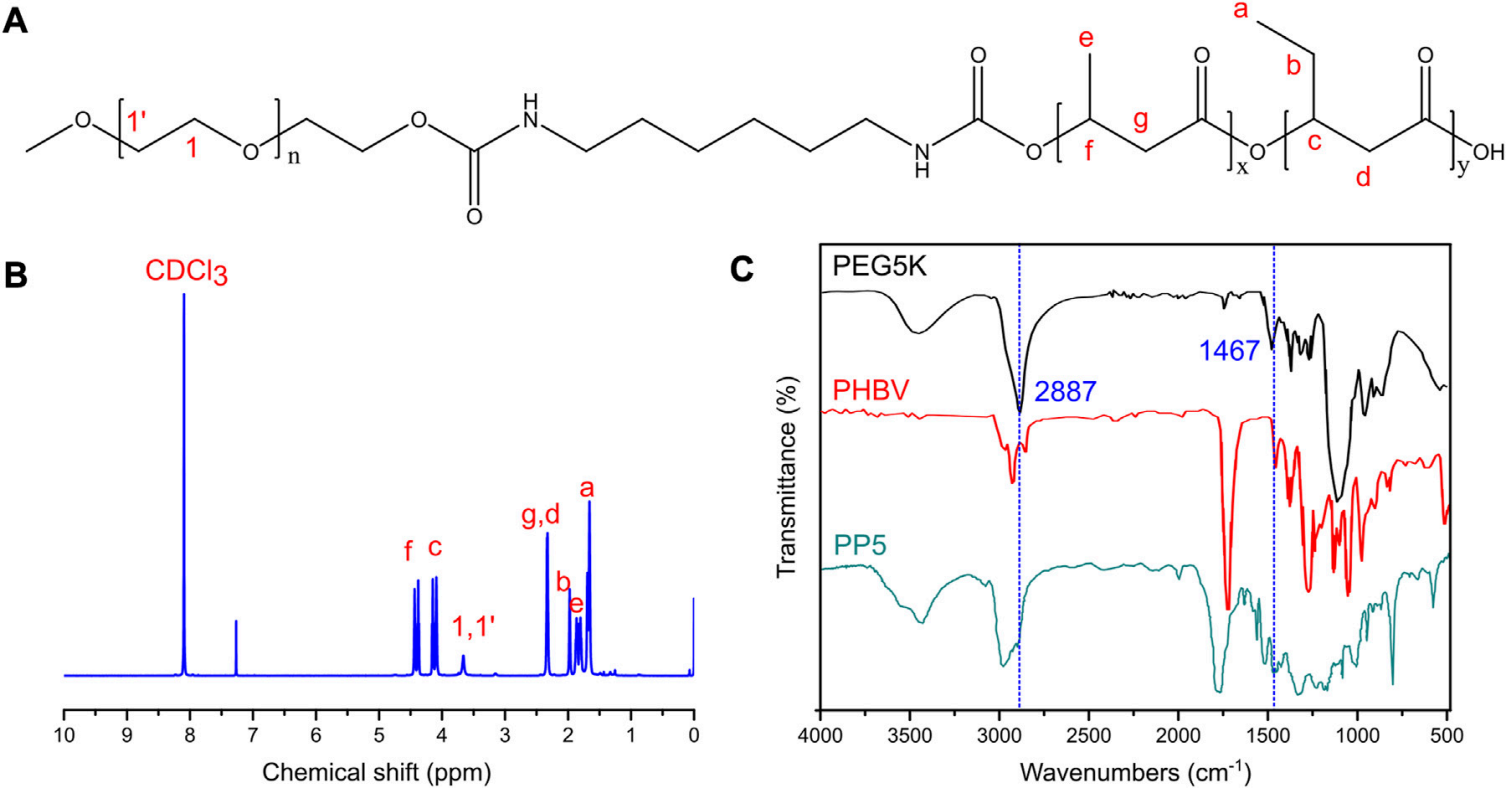
Characteristics of PP5 materials **(A)** The structural formula of PP5. **(B)** The NMR H1 profiles of PP5. **(C)** The FT-IR of PEG5K, PHBV and PP5.

In order to overcome the limitations of PHBV, we opted to synthesize PP5 using PBHV and mPEG5k as the primary raw materials. The chemical structure of PP5 was confirmed through 1H NMR analysis as depicted in Figure 1, *δ* = 1.5−2.03, 2.32,4.0−4.19,4.32−4.47 ppm belongs to PHBV (a-g) while *δ* = 3.66 ppm was corresponded to mPEG5k, which confirmed that we graft mPEG5k to PHBV successfully (Park et al., 1998; Pasek-Allen et al., 2023). The structure of PEG5k, PHBV and PP5 were characterized by FT-IR (Figure 1). In the spectrum of mPEG5k, the absorption peak at approximately at 2,887 and 1,467 cm^−1^ respectively are associated with the stretching and bending vibration of methylene (Xiang et al., 2013). And the peaks also appear in spectrum of PP5, therefore, the FT-IR results confirm that PP5 has been produced.

### 3.2 Biocompatibility of PP5

**FIGURE 2:**
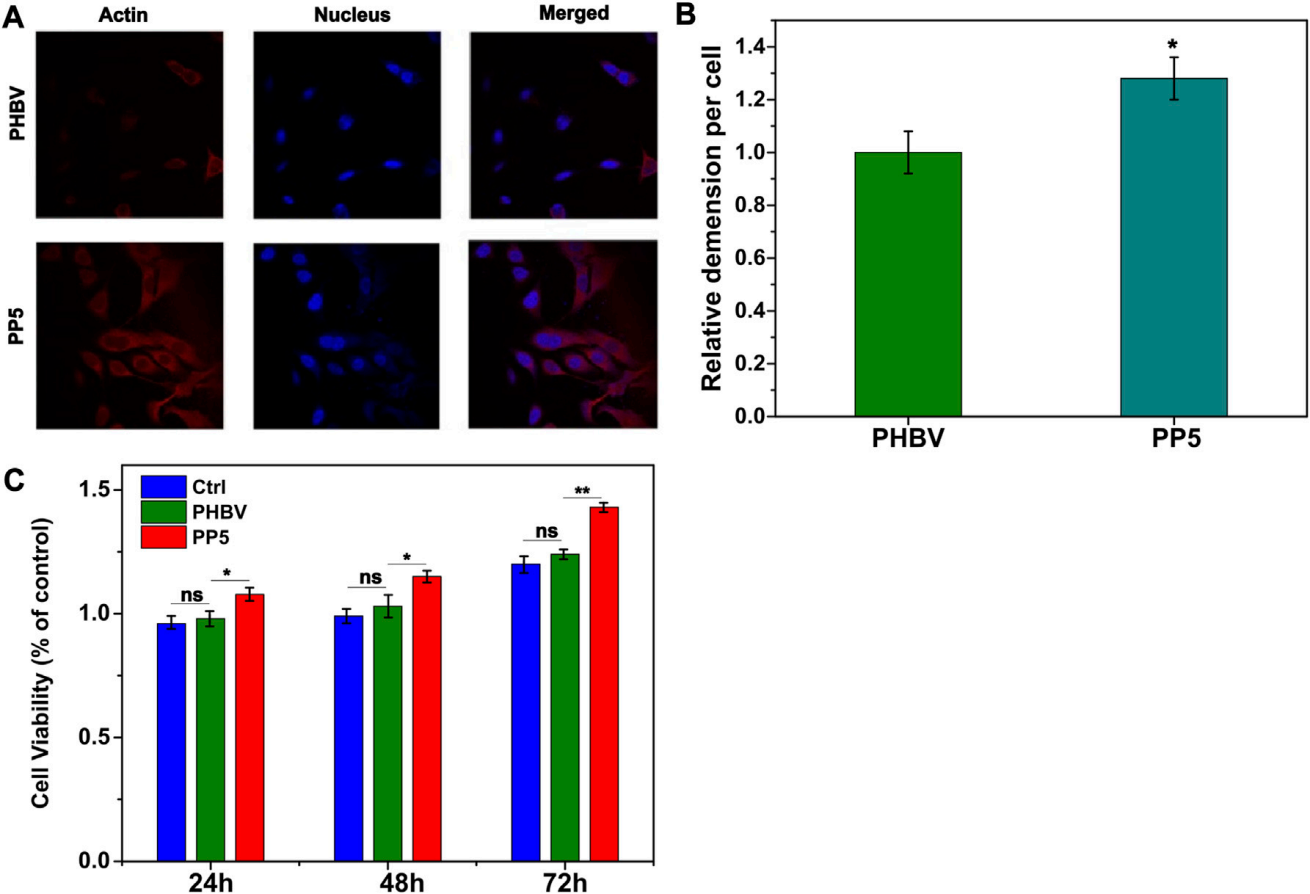
Biocompatibility of PP5 materials. **(A)** Fluorescence observation of cell ductility cocultured with PHBV and PP5 materials; **(B)** The proliferation of HBMSCs co-cultured with PBHV and PP5. **(C)** Cell viability at different exposure times (24 h, 48 h, and 72 h) cocultured with PHBV, PP5 materials and Ctrl.

We utilized the material-cell co-culture method to evaluate the cytotoxicity of PBHV and PP5 and determine their biocompatibility. Human bone marrow mesenchymal cells were co-cultured with the materials for 24, 48, and 72 h, and cell viability was assessed using the CCK8 assay kit. Our findings indicated that both materials displayed no-cytotoxicity and showed some pro-proliferative effect. Moreover, PP5 exhibited a more pronounced pro-proliferative effect compared to PHBV (Figure 2C). Cellular immunofluorescence staining for cytoskeletal proteins (β-tublin) was performed to evaluate cell spreading on the scaffold materials. Fluorescence analysis (Figure 2A) demonstrated that cells exhibited greater extensibility on PP5. In conclusion, the addition with mPEG5k resulted in the synthesis of PP5 biomaterials with improved hydrophilicity and biocompatibility.

### 3.3 Osteotropic effects of PP5 *in vivo* and *in vitro*

**FIGURE 3:**
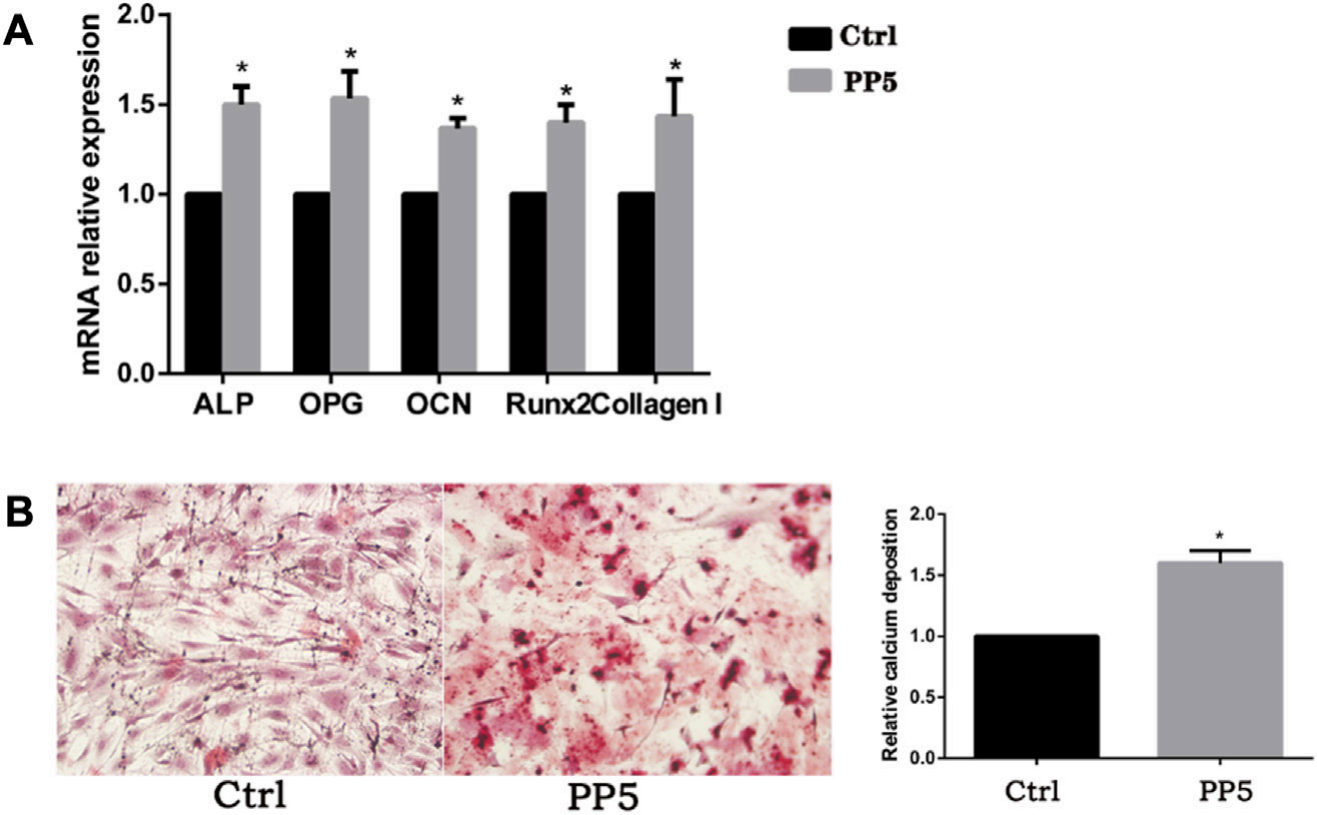
Pharmacokinetics of melatonin. **(A)** UV scanning spectrum of melatonin standard. **(B)** The standard curve of melatonin. **(C)** The drug loading efficiency of melatonin with different GelMA concentration. **(D)** The drug loading capacity of melatonin with different GelMA concentration. **(E)** The drug release rate of melatonin loaded GelMA.

**FIGURE 4:**
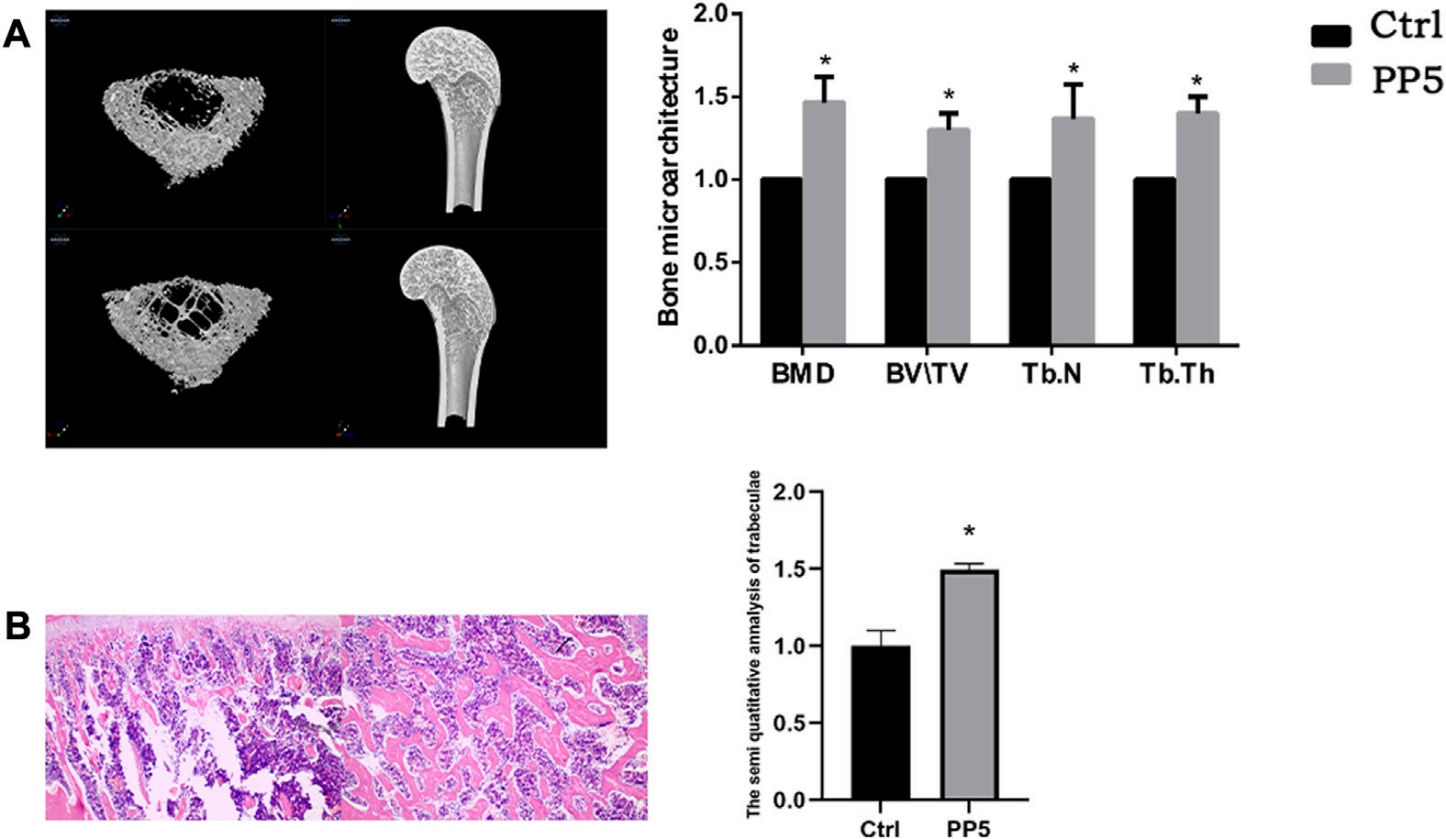
Assessment of *in vitro* osteogenic efficacy of PP5 **(A)** Relative osteogenesis-related gene expressions of the hMSCs cultured on PP5 for 7 days by real-time PCR. **(B)** Alizarin red staining of hMSCs cultured on PP5 and quantitative analysis by ImageJ.

To further evaluate the osteogenic ability of PP5, we conducted both cellular and animal models to investigate the osteogenic effect of each material. We co-cultured both materials with cells and performed RT-PCR analysis of osteogenesis-related genes. (Figure 3A). The expression of osteogenesis-related genes such as ALP, OPG, OCN, Runx2, and Collagen I were significantly higher in the PP5 group compared to the PBHV group. In the alizarin red mineralized nodules staining assay, the PP5 group exhibited higher mineralized nodules compared with control group. For the *in vivo* study, we utilized a femoral defect model in SD rats, a small bone defect was artificially created in the lower femur of SD rats and then implanted with PBHV and PP5, respectively. After euthanasia at 8 weeks postoperatively, the specimens were taken for Micro-CT analysis and HE. As shown in Figure 4A, after data analysis by specific software, the bone mineral density (BMD), bone volume fraction (BV/TV), bone trabecular number (Tb.N), and bone trabecular thickness (Tb.Th) were found to be statistically higher in the PP5 group compared to the PBHV group (*p* < 0.05). These differences were also observed in HE staining (Figure 4B). From the above, it can be concluded that PP5 has a significantly better bone healing promoting effect than PBHV.

### 3.4 Loading and release effects of melatonin on PP5

**FIGURE 5:**
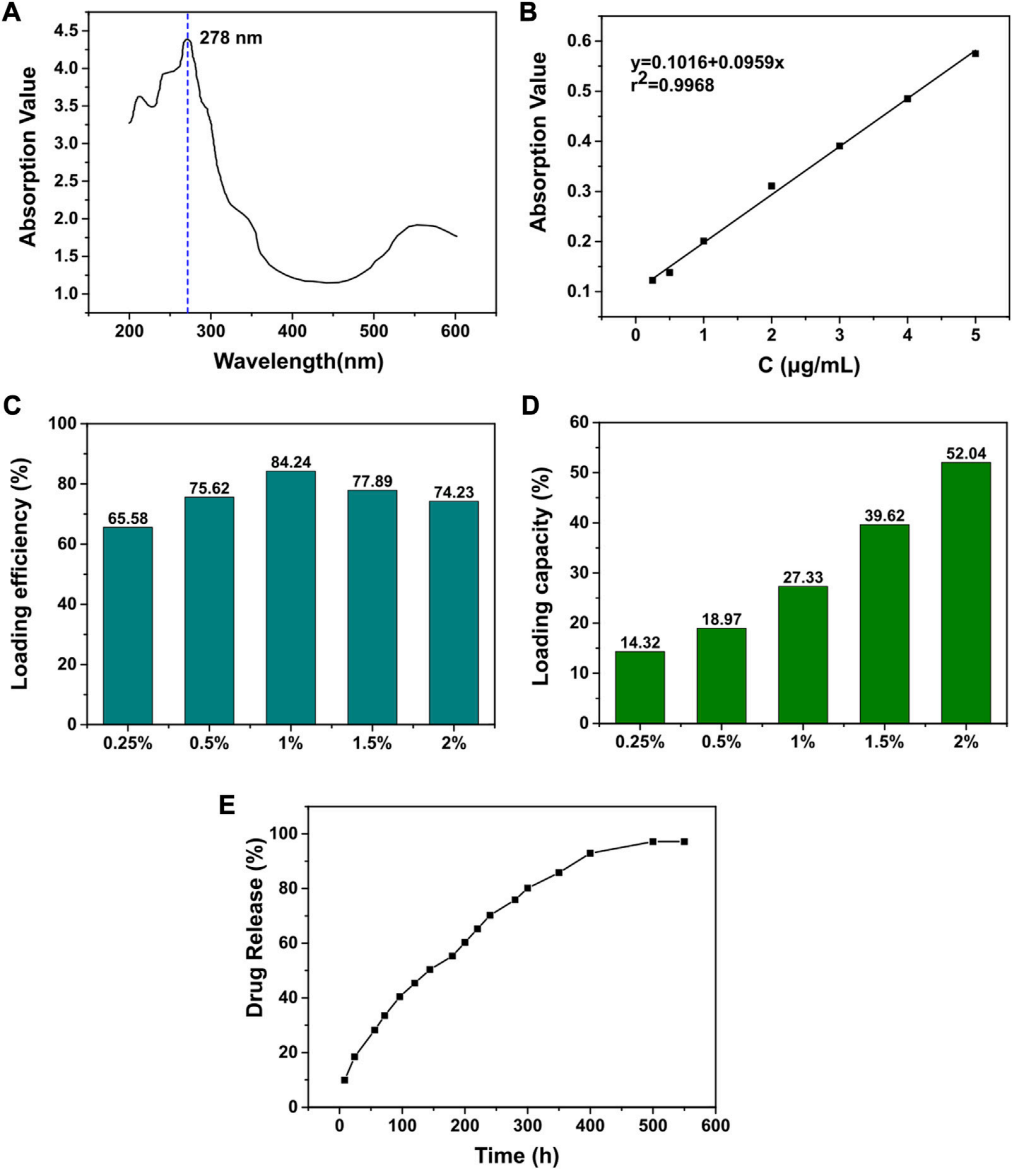
Assessing the *in vivo* osteotropic efficacy of PP5 **(A)** MicroCT evaluation of the effect of PP5 on bone microstructure. **(B)** HE staining to evaluate the promoting effect of PP5 on trabecular bone.

As a potential drug against bone tumors, we proposed to construct a local drug delivery system by loading melatonin to provide a new option for improving the quality of comprehensive management of bone tumors after surgery. Through GelMA, we successfully loaded melatonin on PP5 scaffold and performed release experiments. (Figure 5). The loading capacity increased with decreasing gel concentration, and the loading efficiency initially increased with the increasing concentration and reached a peak at the gel concentration of 1%, so the 1% gel concentration was used for subsequent experiments. In the drug release curve, the melatonin release process was able to sustain the release for more than 14 days without sudden mass release, and the release efficiency of MT was able to reach more than 97.1%. The result indicating that the MT loaded PP5 has the ability to achieve sustained and slow release of MT.

### 3.5 Antitumor capacity of melatonin-loaded drug delivery system (MT + PP5)

**FIGURE 6:**
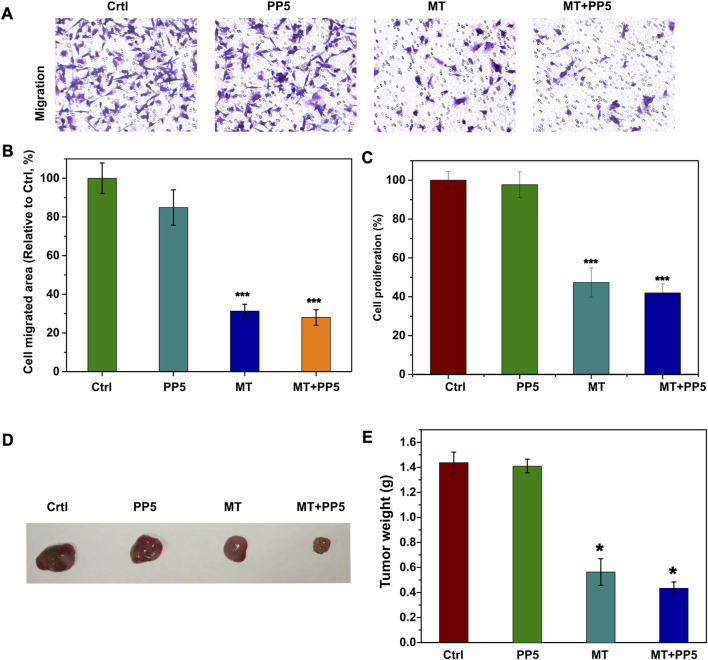
Anti-tumor effect of drug delivery system **(A)** Effect of MT + PP5 on MG63 cell migration *in vitro* and quantitative analysis by ImageJ **(B, C)** Estimation of MG63 cell proliferation on PP5 materials. **(D, E)** Evaluation of tumor suppressive effect of DDS by subcutaneous tumor model.

To evaluate the anti-tumor efficacy of the drug delivery system, we conducted both *in vitro* and *in vivo* experiments. Specifically, we treated tumor cells (MG-3) with two different scaffold and melatonin for a period of 24 h. We then performed cell transwell assays and cell proliferation assays (Figures 6A–C). Both the free melatonin group and MT + PP5 group exhibited anti-bone tumor abilities. In addition, the cell proliferation assay measured by the CCK8 kit also demonstrated inhibitory effects of the above two groups on the proliferation of MG63 cells. Interestingly, the MT + PP5 group showed stronger anti-tumor ability than the free MT group in both experiments.

*In vivo* experiments, we utilized a nude mouse allogeneic subcutaneous tumorigenesis model, once the subcutaneous tumor volume reached 300 mm^2^ in nude mice, we implanted scaffold materials subcutaneously in the tumors, and the tumor specimens were euthanized and weighed after 14 days (Figure 6D). The tumors in the MT + PP5 group were significantly smaller than those in the other three groups.

### 3.6 Study on the mechanism of PP5 to enhance the anti-tumor effect of melatonin

**FIGURE 7:**
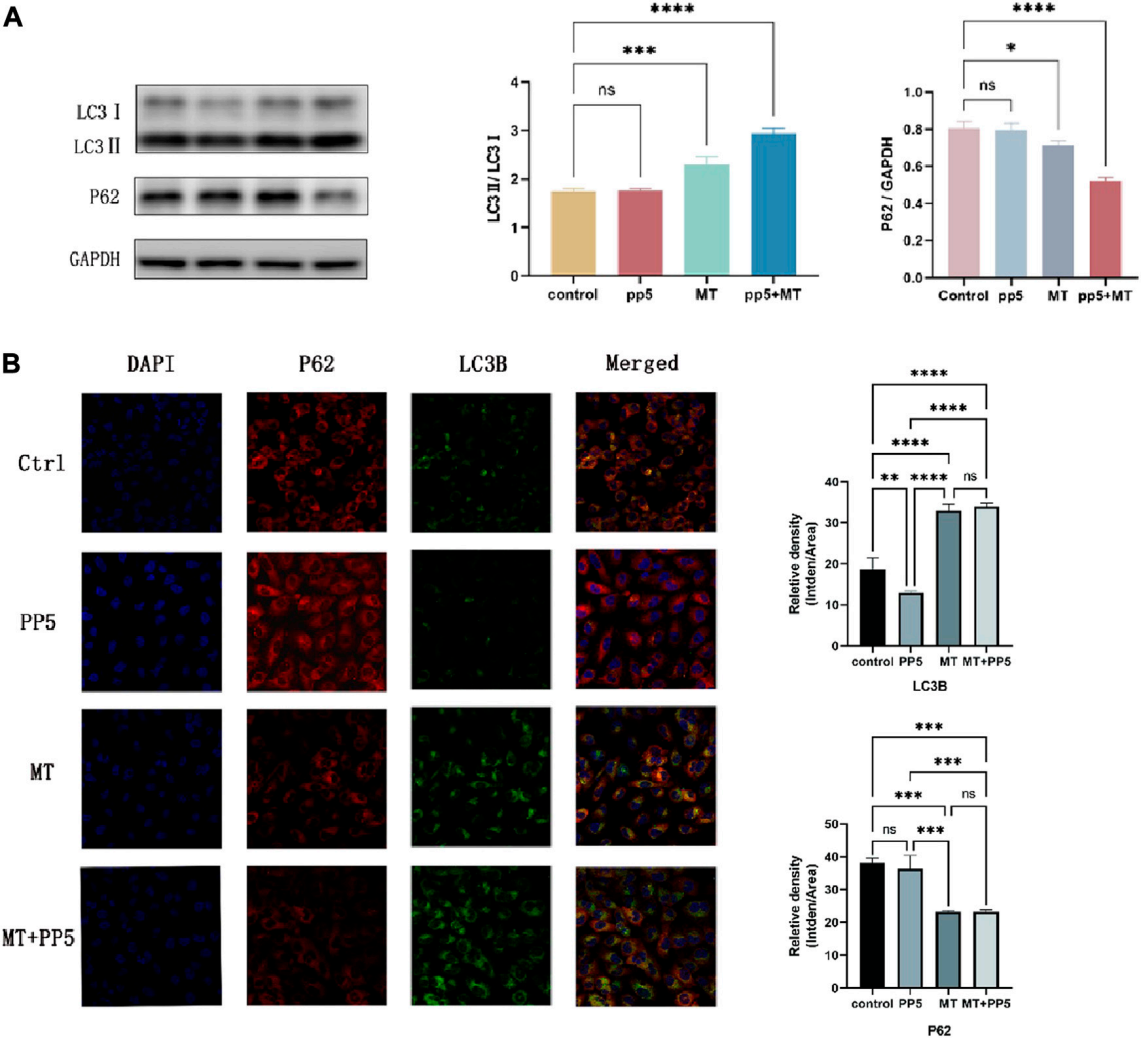
Effect of MT-loaded PP5 scaffold on cellular autophagy **(A)** LC3 and P62 protein expression bands and quantification results after co-culture of tumor cells (MG63) with PP5, MT + PP5, and free melatonin for 48 h, respectively. **(B)** Immunofluorescence staining and quantification of LC3 and P62 after co-culture of tumor cells (MG63) with PP5, MT + PP5, and free melatonin for 48 h, respectively.

To further explore the enhanced antitumor effect of melatonin, we examined the autophagic effect of melatonin on tumors. Western blotting and cellular immunofluorescence showed differential expression of markers associated with cellular autophagy (Figure 7A). The LC3B protein expression was upregulated in the MT group and MT + PP5 group, while P62 protein expression was decreased, with a greater difference observed in the MT + PP5 group. The difference in protein expressions was confirmed through cellular immunofluorescence (Figure 7B). Based on the results, it can be inferred that PP5 can enhance the anti-tumor effects of melatonin by promoting autophagy.

## 4 Discussion

Currently, the clinical treatment of OS involves preoperative or neoadjuvant chemotherapy, surgical removal of primary and metastatic tumor sites, and post-operative chemotherapy. However, surgical resection often results in bone defects that require reconstruction using biomaterials. The biodegradable material poly (3-hydroxybutyrate-co-3-hydroxyvalerate) (PHBV) scaffold has good biodegradability, but its application in the biological field is limited due to poor biocompatibility. Further research is necessary to enhance these characteristics. Polyethylene glycol (PEG) is a faculty of water-soluble polymers with various molecular weights that possess beneficial properties such as protein resistance, low toxicity and immunogenicity. Due to their biocompatibility and minimal toxicity and good solubility in water or other common solvents, PEGs are often selected as drug carriers. In this study, we designed and evaluated PP5 scaffold for its potential to promote bone repair. Material characterization experiments were performed to determine the molecular structure of PP5, as shown in Figures 1A, B, *δ* = 1.5–2.03, 2.32, 4.0–4.19, 4.32–4.47 ppm belongs to PHBV (a-g) while *δ* = 3.66 ppm was correspond to mPEG in ^1^H NMR spectrum suggest that we graft mPEG to PHBV successfully. (Park et al., 1998). And the result was also proved in FT-IR, the absorption peak at approximately at 2,887–1,467 cm^-1^ were associated with the stretching and bending vibration of methylene, the result was the same with reported work. In order to evaluate the bioactivity of PP5, the experiments *in vivo* and *in vitro* showed that PP5 exhibited superior biocompatibility and anti-tumor ability than PHBV, in Figure 2B, C, the cell dimension cultured on PP5 is about 1.35 times than PHBV, the cell viability is significantly more than PHBV and control group especially at 72 h. Also, the expression of ALP, OPG, OCN, Runx2 and Collagen I of PP5 group were significantly higher than Control group, indicated that PP5 scaffold could promote bone growth and might be a suitable option for bone tissue engineering; however, it cannot inhibit tumor metastasis.

At present, the clinical treatment mode of OS is utilizing combination of surgical intervention and chemo/radiotherapy. Inevitably, surgical resection can result in extensive bone defects and leave behind residual neoplastic cells in the defect area, increasing the risk of local recurrence. However, the postsurgical chemo/radiotherapy still encounters some drawbacks, such as inefficient elimination of the residual cancer cells and severe side effects. Meanwhile, the extensive bone defect, the low immunity caused by chemo/radiotherapy, infection and poor blood supply can cause serious issues for defect healing. Melatonin, as a hormone naturally produced in the human body, has the natural advantages of good biocompatibility and high safety. Studies have shown that melatonin can inhibit tumor growth and metastasis. However, higher doses of melatonin are often necessary to achieve the desired effect of tumor suppression. In contrast to systemic high-dose melatonin, topical application of the drug not only achieves the necessary concentration for treatment, but also mitigates the adverse effects of systemic administration, this is particularly beneficial in adolescents, who are more sensitivity to melatonin.

In this study, we used hydrogel to load melatonin on PP5 scaffold. Our results suggested the cumulative release rate of melatonin has reached 96.3% at 10 h, while the drug release cycle of melatonin loaded GelMA can reach 14 days and there is no obvious burst release (Figure 2). Through anti-tumor experiments *in vivo* and *in vitro*, we found that MT-loaded PP5 scaffold has better anti-tumor effect than melatonin alone, the cell migration in transwell assay showed that the MG-3 cell was inhabited of in the MT and MT-loaded PP5 group in Figrue 6, more interestingly, the MT-loaded PP5 group has more effective anti-tumor ability, the weight of tumor in MT-loaded PP5 group was smaller than other two groups, in order to further analyze this phenomenon, we detected autophagy-related markers, including LC3 and P62. The result was shown in Figure 7, autophagy in MT + PP5 group exhibited stronger than that in MT group. Autophagy is a crucial and conserved cellular process that selectively targets abnormal organelles and proteins for lysosomal degradation. The role of autophagy in cancer is controversial. Autophagy can act as a cytoprotective response to chemotherapeutic drugs in cancer cells, and it can also promote metastasis by facilitating the mobility and anoikic resistance of tumor cells. However, several studies have shown that autophagy can induce autophagic cell death, inhibit cell proliferation, and degrade oncoproteins. This can suppress tumorigenesis, impede metastasis, and even enhance chemosensitivity. Several studies have shown that melatonin has antitumor effects, but the exact mechanism is still unknown. Our study found that melatonin induces enhanced autophagy in tumor cells and induces autophagic cell death, resulting in anti-tumorigenic and metastatic effects. The MT-loaded PP5 drug delivery system showed stronger autophagic effect, which seems to be attributed to the slow release of melatonin with local concentration above the therapeutic threshold, the MT group did have significant autophagic effect, but without sustain release ability, MT will be diffused and metabolized in a short time, so the autophagic effect is limited. However, the detailed mechanism needs to be further investigated.

## 5 Conclusion

In summary, a bifunctional scaffold which can inhibit bone tumor recurrence and enhance bone regeneration has been successfully developed by coating MT-loading GelMA on porous surfaces of freeze-dried PP5 prepared with PHBV and mPEG5k by a novel method. The introduction of mPEG5k significantly improves the bio-activity of PP5 scaffold, which leads to the great bone regeneration capacity. The GelMA coating realize long-acting and slowly release of MT from scaffold and efficiently improves the ant-tumor effect. This material meets the necessary requirements for promoting bone repair and eliminating residual tumor to prevent recurrence and metastasis. Therefore, it provides a promising option to improve the comprehensive treatment of bone tumors after surgery. Additionally, due to its excellent ability to promote bone regeneration, this approach is also suitable for treating bone defect diseases or other conditions that require bone implantation.

## Data Availability

The original contributions presented in the study are included in the article/Supplementary Material, further inquiries can be directed to the corresponding author.
